# The G Protein-Coupled Receptor FFAR2 Promotes Internalization during Influenza A Virus Entry

**DOI:** 10.1128/JVI.01707-19

**Published:** 2020-01-06

**Authors:** Guangwen Wang, Li Jiang, Jinliang Wang, Jie Zhang, Fandi Kong, Qibing Li, Ya Yan, Shanyu Huang, Yuhui Zhao, Libin Liang, Junping Li, Nan Sun, Yuzhen Hu, Wenjun Shi, Guohua Deng, Pucheng Chen, Liling Liu, Xianying Zeng, Guobin Tian, Zhigao Bu, Hualan Chen, Chengjun Li

**Affiliations:** a State Key Laboratory of Veterinary Biotechnology, Harbin Veterinary Research Institute, Chinese Academy of Agricultural Sciences, Harbin, China; University of North Carolina at Chapel Hill

**Keywords:** β-arrestin1, AP2B1, FFAR2, GRK, entry, influenza A virus, internalization

## Abstract

Influenza A virus (IAV) coopts numerous host factors to complete its replication cycle. Here, we identify free fatty acid receptor 2 (FFAR2) as a cofactor for IAV entry into host cells. We found that downregulation of FFAR2 or Ffar2 expression significantly reduced the replication of IAV in A549 or RAW 264.7 cells. The treatment of A549 cells with small interfering RNA (siRNA) targeting *FFAR2* or the FFAR2 pathway agonists 2-(4-chlorophenyl)-3-methyl-*N*-(thiazol-2-yl)butanamide (4-CMTB) and compound 58 (Cmp58) [(*S*)-2-(4-chlorophenyl)-3,3-dimethyl-*N*-(5-phenylthiazol-2-yl)butanamide] dramatically inhibited the nuclear accumulation of viral nucleoprotein (NP) at early time points postinfection, indicating that FFAR2 functions in the early stage of the IAV replication cycle. FFAR2 downregulation had no effect on the expression of sialic acid (SA) receptors on the cell membrane, the attachment of IAV to the SA receptors, or the activity of the viral ribonucleoprotein (vRNP) complex. Rather, the amount of internalized IAVs was significantly reduced in FFAR2-knocked-down or 4-CMTB- or Cmp58-treated A549 cells. Further studies showed that FFAR2 associated with β-arrestin1 and that β-arrestin1 interacted with the β2-subunit of the AP-2 complex (AP2B1), the essential adaptor of the clathrin-mediated endocytosis pathway. Notably, siRNA knockdown of either β-arrestin1 or AP2B1 dramatically impaired IAV replication, and AP2B1 knockdown or treatment with Barbadin, an inhibitor targeting the β-arrestin1/AP2B1 complex, remarkably decreased the amount of internalized IAVs. Moreover, we found that FFAR2 interacted with three G protein-coupled receptor (GPCR) kinases (i.e., GRK2, GRK5, and GRK6) whose downregulation inhibited IAV replication. Together, our findings demonstrate that the FFAR2 signaling cascade is important for the efficient endocytosis of IAV into host cells.

**IMPORTANCE** To complete its replication cycle, IAV hijacks the host endocytosis machinery to invade cells. However, the underlying mechanisms of how IAV is internalized into host cells remain poorly understood, emphasizing the need to elucidate the role of host factors in IAV entry into cells. In this study, we identified FFAR2 as an important host factor for the efficient replication of both low-pathogenic and highly pathogenic IAV. We revealed that FFAR2 facilitates the internalization of IAV into target cells during the early stage of infection. Upon further characterization of the role of FFAR2-associated proteins in virus replication, we found that the FFAR2–β-arrestin1–AP2B1 signaling cascade is important for the efficient endocytosis of IAV. Our findings thus further our understanding of the biological details of IAV entry into host cells and establish FFAR2 as a potential target for antiviral drug development.

## Abstract (precis)

To complete its replication cycle, IAV hijacks the host endocytosis machinery to invade cells. However, the underlying mechanisms of how IAV is internalized into host cells remain poorly understood, emphasizing the need to elucidate the role of host factors in IAV entry into cells. In this study, we identified FFAR2 as an important host factor for the efficient replication of both low-pathogenic and highly pathogenic IAV. We revealed that FFAR2 facilitates the internalization of IAV into target cells during the early stage of infection. Upon further characterization of the role of FFAR2-associated proteins in virus replication, we found that the FFAR2–β-arrestin1–AP2B1 signaling cascade is important for the efficient endocytosis of IAV. Our findings thus further our understanding of the biological details of IAV entry into host cells and establish FFAR2 as a potential target for antiviral drug development.

## INTRODUCTION

Influenza A virus (IAV) is an important pathogen of the family *Orthomyxoviridae*. Aside from frequent seasonal epidemics caused by H1 or H3 IAVs and occasional pandemics, avian influenza viruses, such as those of the H5N1, H7N9, and H9N2 subtypes, are currently posing increasing threats to humans (1–6). The genome of IAV consists of eight single-stranded negative-sense RNA segments that encode 10 essential proteins: polymerase basic protein 2 (PB2), polymerase basic protein 1 (PB1), polymerase acidic protein (PA), hemagglutinin (HA), nucleoprotein (NP), neuraminidase (NA), matrix protein 1 (M1), matrix protein 2 (M2), nonstructural protein 1 (NS1), and NS2. Of these viral proteins, HA is responsible for mediating the binding of virus particles to sialic acid (SA) receptors on cell surface glycoproteins or glycolipids (7), which is the initial step for IAV entry into host cells.

After binding to SA receptors, IAV is internalized by receptor-mediated endocytosis. Rust et al. previously demonstrated that most IAV particles enter cells through clathrin-mediated endocytosis (CME) (8). Besides, IAV is also internalized through caveola-mediated endocytosis, clathrin- and caveola-independent endocytosis, and macropinocytosis (9, 10). Many cellular factors have been implicated in IAV entry because HA binding to SA receptors alone is insufficient for successful viral endocytosis into host cells. For example, EPN1 is recruited to the binding sites of IAV, where it initiates the formation of clathrin-coated pits, ultimately resulting in the internalization of the virus (11). Loss of terminal N-linked glycosylation due to a mutation in the *GNT1* gene arrests IAV internalization at the plasma membrane before the virus is endocytosed (12). Plasma membrane lipid raft clustering, caused by IAV attachment, activates epidermal growth factor receptor (EGFR) (13), leading to the activation of downstream signaling molecules, such as phosphatidylinositol 3-kinase (PI3K) and phospholipase C γ1 (PLC-γ1) (14, 15), which enhances IAV uptake. CD81 promotes the trafficking of IAV to fusion-competent endosomes and further organizes the endosomal membrane to assist viral fusion (16). Upon IAV infection, Itch is phosphorylated and recruited to endosomes, where it ubiquitinates the viral M1 protein and mediates the release of viral ribonucleoprotein (vRNP) complexes from the endosomal compartments (17). Although these cellular factors have been implicated in IAV endocytosis, the biological details underlying the IAV entry process remain poorly understood, and the roles of other host factors have yet to be revealed.

G protein-coupled receptor (GPCR), a seven-α-helix transmembrane segment receptor, represents the largest superfamily of cell surface receptors and regulates a large array of biological functions (18). Roles for GPCR family members in the replication of different viruses have been increasingly demonstrated. Notably, CCR5 and CXCR4 are required for HIV-1 infectivity, acting as coreceptors of the viral envelope glycoprotein gp120 (19), and metabotropic glutamate receptor 2 (mGluR2) is a novel cellular receptor for rabies virus (RABV) through interaction with RABV G protein (20). GPCR antagonists targeting histamine receptors, 5-hydroxytryptamine (5-HT) (serotonin) receptors, muscarinic acetylcholine receptor, and adrenergic receptor block the entry of Ebola virus and Marburg virus at a step that follows initial attachment but prior to viral/cell membrane fusion (21). GPCR proteins are also involved in the replication and pathogenesis of IAV. It has been reported that stimulation of α2-adrenergic receptors by clonidine inhibits IAV replication (22), and treatment of mice with the angiotensin II inhibitor losartan alleviates lung edema and improves lung histopathology, although the viral load in the lung tissue of mice is not reduced (23).

Free fatty acid receptor 2 (FFAR2) (also known as GPR43), together with FFAR1 and FFAR3, is classified as a rhodopsin-like receptor and clusters at chromosome 19q13.1 in humans (24). *FFAR2* mRNA is highly expressed in immune cells such as monocytes, neutrophils (25, 26), dendritic cells (27), and regulatory T cells (28). FFAR2 can be activated by short-chain fatty acids such as acetate and propionate (29, 30), and this activation is coupled to inositol 1,4,5-trisphosphate formation, intracellular Ca^2+^ release, extracellular signal-regulated kinase 1/2 (ERK1/2) activation, inhibition of cAMP accumulation (29, 31), and modulation of the p38, Jun N-terminal protein kinase (JNK), and Akt signaling pathways (32, 33). FFAR2 has also been linked to the severity of inflammation, although different studies have reached contentious conclusions (28, 34–37). However, a role for FFAR2 in virus infection has never been demonstrated.

In the present study, we demonstrate that FFAR2 is a novel host factor for the efficient replication of IAV and discover that FFAR2 plays an important role in the entry step of the virus life cycle. We further found that FFAR2-mediated IAV internalization involves downstream signaling molecules such as G protein-coupled receptor kinases (GRKs), β-arrestin1, and the AP-2 complex.

## RESULTS

### FFAR2 is important for infection by different subtypes of IAV.

**FIG 1:**
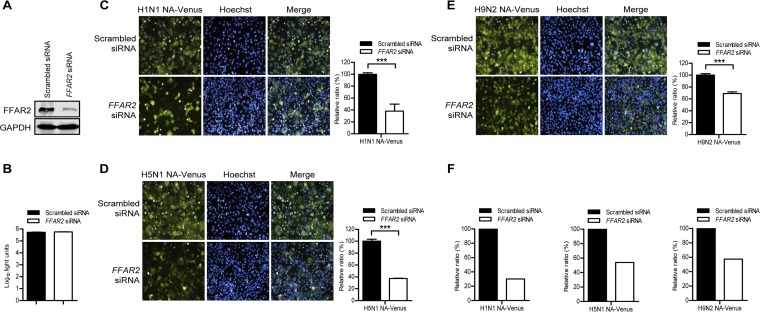
Identification of FFAR2 as a host factor involved in the replication of IAV by using H1N1 NA-Venus, H5N1 NA-Venus, and H9N2 NA-Venus reporter viruses. (A) A549 cells were transfected with siRNA targeting *FFAR2* or with scrambled siRNA for 48 h, and the knockdown of FFAR2 was detected by Western blotting. (B) A549 cells were treated with *FFAR2* siRNA or scrambled siRNA and cultured at 37°C for 48 h. Cell viability was determined by using a CellTiter-Glo assay. (C to E) A549 cells were seeded into 96-well plates containing *FFAR2* siRNA or scrambled siRNA and cultured at 37°C for 48 h. They were then infected with one of the Venus reporter viruses, H1N1 NA-Venus (MOI = 0.1) (C), H5N1 NA-Venus (MOI = 0.1) (D), or H9N2 NA-Venus (MOI = 0.1) (E), and cultured at 37°C for 24 h. Venus expression was visualized by using the Operetta high-content imaging system. The effect of FFAR2 knockdown by siRNA interference on virus replication was calculated by normalizing the average fluorescence intensity of the *FFAR2* siRNA-treated wells with that of the scrambled siRNA-treated cells. The data are presented as means ± standard deviations (SD) for triplicate samples. ***, *P < *0.001. (F) A549 cells were infected as described above for panels C to E, and viral NP was detected by immunostaining. The cell nuclei were stained with Hoechst 33342. The blue and red fluorescence images were collected from the same area and used to evaluate the effect of FFAR2 knockdown on the replication of H1N1 NA-Venus, H5N1 NA-Venus, or H9N2 NA-Venus by quantifying the percentage of infected cells as follows: number of cells expressing NP protein (red)/total number of cells (blue).

We identified FFAR2 as a potential host factor for the replication of IAV by using a whole-genome small interfering RNA (siRNA) library screen (our unpublished data) targeting 21,585 mRNAs and a replication-competent Venus-expressing H5N1 virus (H5N1 NA-Venus) (38). To confirm this finding, we analyzed the impact of siRNA-mediated FFAR2 knockdown on the growth of different reporter viruses expressing Venus fluorescent protein, namely, H1N1 NA-Venus, H5N1 NA-Venus, and H9N2 NA-Venus viruses. We found that *FFAR2* siRNA treatment efficiently reduced the expression of FFAR2 without adversely affecting cell viability (Fig. 1A and B). At 24 h postinfection (p.i.), the fluorescence intensity of the *FFAR2* siRNA-treated A549 cells was normalized to that of the scrambled siRNA-treated cells. FFAR2 downregulation by siRNA silencing produced at least a 30% reduction in fluorescence intensity in the cells infected with H1N1 NA-Venus, H5N1 NA-Venus, or H9N2 NA-Venus virus (Fig. 1C to E). The inhibitory effect of FFAR2 knockdown on the growth of the NA-Venus reporter viruses was also apparent when NP staining was used as an indicator to quantify the percentage of infected cells (Fig. 1F).

**FIG 2:**
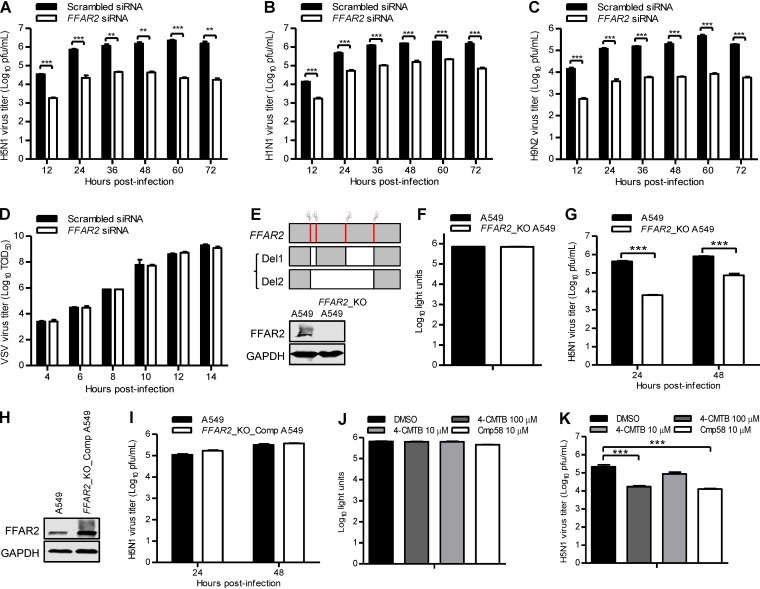
FFAR2 positively regulates IAV replication in A549 cells. (A to D) *FFAR2* siRNA- or scrambled siRNA-transfected A549 cells were infected with AH05 (H5N1) (MOI = 0.1) (A), WSN (H1N1) (MOI = 0.01) (B), SH13 (H9N2) (MOI = 0.1) (C), or VSV-EGFP (100 TCID_50_) (D) virus. Supernatants were collected at the indicated time points, and virus titers were determined by means of plaque assays on MDCK cells (A to C) or by determining the TCID_50_ on HEK293T cells (D). **, *P < *0.01; ***, *P < *0.001. (E) Schematic diagram of four sgRNA targeting sites at the *FFAR2* gene loci and the two kinds of *FFAR2* truncated mutants. Sanger sequencing results indicated that a frameshift occurred in the *FFAR2*-truncated mutants within the *FFAR2*_KO cell line; the knockout of *FFAR2* was confirmed by Western blotting. (F) Cell viability of *FFAR2*_KO A549 cells was measured by using the CellTiter-Glo assay. (G) AH05 (H5N1) virus replication in *FFAR2*_KO A549 cells. *FFAR2*_KO A549 cells or control cells were infected with AH05 (H5N1) (MOI = 0.1). Supernatants were collected at 24 and 48 h p.i., and virus titers were determined by means of plaque assays on MDCK cells. ***, *P < *0.001. (H) The expression of FFAR2 in *FFAR2*_KO_Comp A549 cells was confirmed by Western blotting. (I) AH05 (H5N1) virus replication in *FFAR2*_KO_Comp A549 cells. *FFAR2*_KO_Comp A549 cells or control A549 cells were infected with AH05 (H5N1) (MOI = 0.1). Supernatants were collected at 24 and 48 h p.i., and virus titers were determined by means of plaque assays on MDCK cells. (J) Cell viability of A549 cells treated with 4-CMTB [2-(4-chlorophenyl)-3-methyl-*N*-(thiazol-2-yl)butanamide] or Cmp58 [(*S*)-2-(4-chlorophenyl)-3,3-dimethyl-*N*-(5-phenylthiazol-2-yl)butanamide] was measured by using the CellTiter-Glo assay. (K) A549 cells were pretreated with 4-CMTB or Cmp58 at the indicated concentrations for 3 h and then infected with AH05 (H5N1) virus (MOI = 0.1). Supernatants were collected at 24 h p.i., and virus titers were determined by means of plaque assays on MDCK cells. ***, *P < *0.001.

The effect of siRNA knockdown of FFAR2 on IAV infection was then evaluated with wild-type viruses. We found that FFAR2 downregulation in A549 cells led to 19- to 100-, 8- to 39-, and 26- to 56-fold reductions in growth titers from 12 to 72 h p.i. for A/Anhui/2/2005 (AH05) (H5N1), A/WSN/1933 (WSN) (H1N1), and A/chicken/Shanghai/SC197/2013 (SH13) (H9N2) viruses, respectively, demonstrating that FFAR2 is critical for the efficient replication of different subtypes of IAV (Fig. 2A to C). To evaluate whether FFAR2 is specifically important for the replication of IAV, we analyzed the effect of FFAR2 knockdown on the replication of vesicular stomatitis virus encoding an enhanced green fluorescent protein reporter gene (VSV-EGFP). We found that FFAR2 knockdown had no effect on the replication titers of VSV-EGFP at the indicated time points (Fig. 2D), thereby demonstrating the specificity of FFAR2 in supporting the replication of IAV. We also generated an *FFAR2* knockout (*FFAR2*_KO) A549 cell line by using the CRISPR/Cas9 system. By simultaneous electrotransfection of four pSpCas9(BB)-2A-GFP (pX458) constructs containing single guide RNA (sgRNA) targeting *FFAR2* to A549 cells, a mixture of knockout cells was successfully generated. Sequencing analysis revealed a 271- or 578-bp deletion compared with the *FFAR2* mRNA sequence (Fig. 2E). Both deletions led to a frameshift in the *FFAR2* alleles that resulted in the loss of FFAR2 expression (Fig. 2E). *FFAR2* knockout had no major effect on cell viability as measured by a luminescent cell viability assay (Fig. 2F). The titers of AH05 (H5N1) virus produced from *FFAR2*_KO A549 cells were dramatically lower than those of the control cells at 24 or 48 h p.i. (Fig. 2G). Moreover, we employed a retroviral packaging system to complement the expression of FFAR2 protein in *FFAR2*_KO A549 cells (Fig. 2H). As shown in Fig. 2I, the complement of FFAR2 protein restored the replication of AH05 (H5N1) virus. These data further demonstrate that FFAR2 positively regulates IAV replication.

The role of FFAR2 in IAV replication was also explored by using 4-CMTB and compound 58 (Cmp58) [(*S*)-2-(4-chlorophenyl)-3,3-dimethyl-*N*-(5-phenylthiazol-2-yl)butanamide], which are allosteric agonists of FFAR2 (39, 40). Treatment with 100 or 10 μM 4-CMTB and 10 μM Cmp58 had no adverse effect on the viability of A549 cells (Fig. 2J). As shown in Fig. 2K, treatment with 100 μM 4-CMTB led to a 13-fold decrease in the AH05 (H5N1) virus titer at 24 h p.i. compared with that in dimethyl sulfoxide (DMSO)-treated cells, whereas a slight inhibitory effect on virus replication was observed in response to treatment with 10 μM 4-CMTB, indicating that 4-CMTB inhibited IAV replication in a dose-dependent manner. Moreover, treatment with 10 μM Cmp58 reduced the viral titer by 17-fold at 24 h p.i. (Fig. 2K). These results also demonstrate that the function of FFAR2 is important for IAV replication.

**FIG 3:**
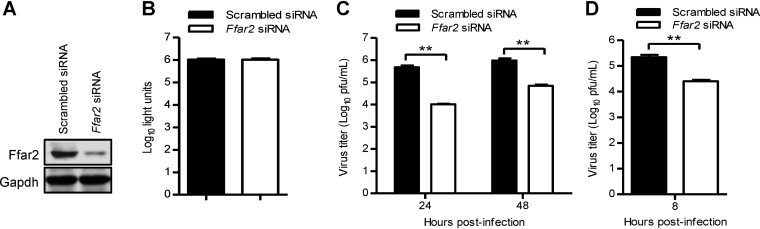
Ffar2 enhances IAV replication in mouse RAW 264.7 cells. (A) Mouse RAW 264.7 cells were transfected with mouse *Ffar2* siRNA or scrambled siRNA for 48 h, and the knockdown of Ffar2 was confirmed by Western blotting with a rabbit anti-FFAR2 pAb. (B) Mouse RAW 264.7 cells were treated with mouse *Ffar2* siRNA or scrambled siRNA and cultured at 37°C for 48 h. Cell viability was determined by using a CellTiter-Glo assay. (C and D) *Ffar2* siRNA- or scrambled siRNA-transfected RAW 264.7 cells were infected with AH05 (H5N1) virus at an MOI of 0.1 (C) or 5 (D). Supernatants were collected at the indicated time points, and virus titers were determined by means of plaque assays on MDCK cells. **, *P < *0.01.

We further evaluated the role of Ffar2 in IAV replication in mouse RAW 264.7 cells. Western blotting showed that the expression of Ffar2 was reduced in specific siRNA-treated RAW 264.7 cells but not in cells treated with scrambled siRNA (Fig. 3A). siRNA treatment targeting *Ffar2* had no cytotoxic effects on RAW 264.7 cells (Fig. 3B), as measured by using a luminescent cell viability assay. Strikingly, 46- and 14-fold decreases in virus titers were observed at 24 and 48 h p.i., respectively, in *Ffar2* siRNA-treated RAW 264.7 cells infected with AH05 (H5N1) virus at a multiplicity of infection (MOI) of 0.1 (Fig. 3C). In addition, *Ffar2* siRNA treatment led to an 8-fold reduction in the virus titer at 8 h p.i. when the cells were infected at an MOI of 5 (Fig. 3D), indicating that Ffar2 is also critical for a single round of IAV replication. Together, our findings show that FFAR2 is engaged in the replication of IAV in different cell types.

**FIG 4:**
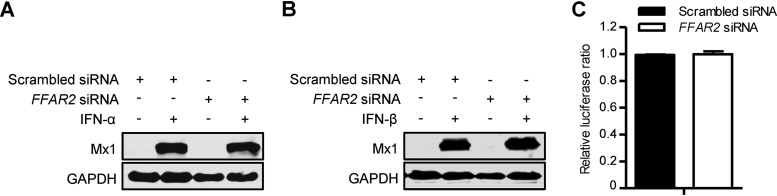
FFAR2 knockdown does not stimulate IFN pathways. (A and B) *FFAR2* siRNA- or scrambled siRNA-transfected A549 cells were left untreated or were treated with IFN-α (A) or IFN-β (B) for 24 h. The cell lysates were then subjected to Western blotting with a rabbit anti-Mx1 pAb for the detection of Mx1 protein. (C) HEK293T cells were treated with *FFAR2* siRNA or scrambled siRNA for 24 h and then transfected with the ISRE-Luc reporter plasmid and the pRL-TK control plasmid. Twenty-four hours later, a dual-luciferase reporter assay was performed. After normalization with cotransfected *Renilla* luciferase activity, the relative firefly luciferase activity of the FFAR2 knockdown cells was expressed as the fold induction of the ISRE firefly luciferase activity compared to control cells.

Stimulation of innate immunity can potently inhibit the replication of IAV. To determine whether the dramatic decrease in virus replication upon downregulation of FFAR2 is associated with the stimulation of interferon (IFN) signaling, we examined the expression of Mx1 protein, a key antiviral effector of the IFN pathway, in *FFAR2* siRNA- or scrambled siRNA-treated A549 cells with or without IFN-α or IFN-β induction. Mx1 was not detectable in cells transfected with siRNA targeting *FFAR2* or scrambled siRNA in the absence of IFN-α or IFN-β stimulation (Fig. 4A and B); no difference in the Mx1 expression level induced by IFN-α or IFN-β treatment was seen in cells treated with *FFAR2* siRNA or scrambled siRNA (Fig. 4A and B). We also determined the interferon-stimulated response element luciferase (ISRE-Luc) reporter activity in *FFAR2* siRNA-treated HEK293T cells and found that FFAR2 knockdown did not change the expression of the ISRE luciferase reporter gene compared with that in the control siRNA-treated cells (Fig. 4C). Taken together, these results demonstrate that the restriction of IAV replication in FFAR2-downregulated cells is not associated with the stimulation of the IFN pathway.

### FFAR2 is involved in the early steps of IAV entry.

**FIG 5:**
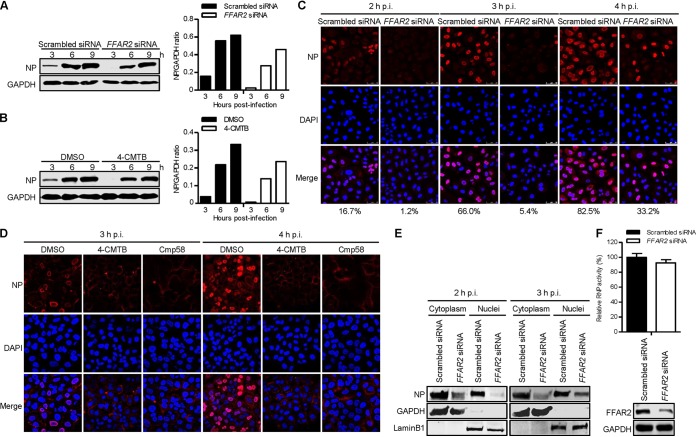
FFAR2 affects the early stage of the IAV replication cycle. (A) *FFAR2* siRNA- or scrambled siRNA-treated A549 cells were infected with AH05 (H5N1) virus (MOI = 5). Whole-cell lysates were collected at the indicated time points and subjected to Western blotting with a rabbit anti-NP pAb. (B) A549 cells were pretreated with 100 μM 4-CMTB or DMSO for 3 h and then infected with AH05 (H5N1) virus (MOI = 5). Whole-cell lysates were collected at the indicated time points and subjected to Western blotting with a rabbit anti-NP pAb. (C) A549 cells were treated with *FFAR2* siRNA or scrambled siRNA for 48 h and then infected with AH05 (H5N1) virus (MOI = 5). At 2, 3, and 4 h p.i., the infected cells were fixed and stained with a rabbit anti-NP pAb, followed by incubation with Alexa Fluor 633 goat anti-rabbit IgG(H+L) (red). The nuclei were stained with DAPI (blue). The ratio of virus-infected cells showing nuclear localization of viral NP was calculated from more than 150 cells and is indicated at the bottom of each panel of three images. (D) A549 cells were pretreated with 100 μM 4-CMTB, 10 μM Cmp58, or DMSO for 3 h and then infected with AH05 (H5N1) virus (MOI = 5). At 3 and 4 h p.i., the infected cells were fixed and stained as described above for panel C. (E) *FFAR2* siRNA- or scrambled siRNA-transfected A549 cells were infected with AH05 (H5N1) virus (MOI = 5). At 2 and 3 h p.i., the cells were separated into nuclear and cytoplasmic fractions. Each fraction was subjected to Western blotting with a rabbit anti-NP pAb, a rabbit anti-GAPDH pAb, and a rabbit anti-LaminB1 pAb. (F) HEK293T cells treated with *FFAR2* siRNA or scrambled siRNA for 24 h were transfected with the four viral RNP protein expression plasmids (PB2, PB1, PA, and NP), together with pHH21-SC09NS F-Luc and pRL-TK. Thirty-six hours later, the knockdown of FFAR2 was confirmed by Western blotting, and a dual-luciferase assay was performed in which the firefly luciferase activity was normalized to the activity of the internal control, *Renilla* luciferase. The band intensities of the Western blots were quantified by using ImageJ software and are expressed as relative NP/GAPDH ratios (A and B).

To investigate the step of the IAV replication cycle in which FFAR2 is engaged, A549 cells knocked down for FFAR2 were infected with AH05 (H5N1) virus at an MOI of 5. At 3, 6, and 9 h p.i., Western blotting showed that viral NP levels in cells silenced with *FFAR2* siRNA were remarkably reduced compared with those in cells treated with scrambled siRNA (Fig. 5A). Moreover, pretreatment of A549 cells with 100 μM 4-CMTB led to significant suppression of viral NP expression at the early time points after infection (Fig. 5B). Notably, the significantly reduced level of NP expression in cells treated with *FFAR2*-specific siRNA or 4-CMTB was observed as early as 3 h p.i., indicating that the host factor FFAR2 was most likely involved in the early steps of IAV replication. To corroborate this finding, we visualized the cellular distribution of NP by means of confocal microscopy in both FFAR2-knocked-down and control A549 cells infected with AH05 (H5N1) virus at early time points of infection. Viral NP was clearly visible in the nucleus of 16.7%, 66.0%, and 82.5% of scrambled siRNA-treated A549 cells at 2, 3, and 4 h p.i., respectively (Fig. 5C). In contrast, only 1.2%, 5.4%, and 33.2% of *FFAR2* siRNA-treated cells displayed clear nuclear accumulation of viral NP at the same time points. These data indicate that FFAR2 downregulation impairs the early stage of IAV replication.

We also examined the cellular distribution of viral NP in A549 cells pretreated with 100 μM 4-CMTB, 10 μM Cmp58, or DMSO. Significantly, pretreatment of cells with 4-CMTB or Cmp58 led to inhibition of NP nuclear accumulation at 3 and 4 h p.i. compared to cells treated with DMSO (Fig. 5D). Therefore, treatment with *FFAR2* siRNA or agonists indicates that the host factor FFAR2 is involved in the early stage of IAV replication.

Next, we validated the inhibitory effect of FFAR2 knockdown on viral NP localization by performing a cell fractionation experiment. *FFAR2* siRNA- and control siRNA-treated A549 cells were infected with AH05 (H5N1) virus at an MOI of 5. At 2 and 3 h p.i., the infected cells were lysed, separated into nuclear and cytoplasmic fractions, and subjected to Western blotting. As shown in Fig. 5E, the marker proteins glyceraldehyde-3-phosphate dehydrogenase (GAPDH) and LaminB1 were detected only in the cytoplasm and nucleus, respectively. Notably, the NP level was markedly reduced in both the cytoplasmic and nuclear extracts of the FFAR2-knocked-down A549 cells compared with those in the control siRNA-treated cells at 2 and 3 h p.i., thereby confirming that FFAR2 downregulation causes a defect in the early stage of virus replication.

We further investigated whether FFAR2 had an effect on the transcription and replication of the IAV genome by using a minigenome replicon assay. *FFAR2* siRNA-treated HEK293T cells were transfected with constructs expressing the four viral RNP proteins (PB2, PB1, PA, and NP) of A/WSN/33 (H1N1) virus, along with a reporter plasmid containing the luciferase gene flanked by the terminal coding and noncoding sequences of the viral NS segment under the control of the human RNA polymerase I promoter and terminator. Thirty-six hours later, the luciferase activity of the cell lysates was determined as a measure of the viral RNP activity. We found that the viral RNP activity remained unchanged when the expression of FFAR2 was knocked down by specific siRNA compared with that in control siRNA-treated cells (Fig. 5F), indicating that the endogenous cellular FFAR2 has no effect on the transcription and replication of the viral genome.

Collectively, these results demonstrate that FFAR2 plays an important role in an early step of the virus replication cycle but not in the transcription or replication of the viral genome.

### FFAR2 is not required for attachment but is important for internalization.

**FIG 6:**
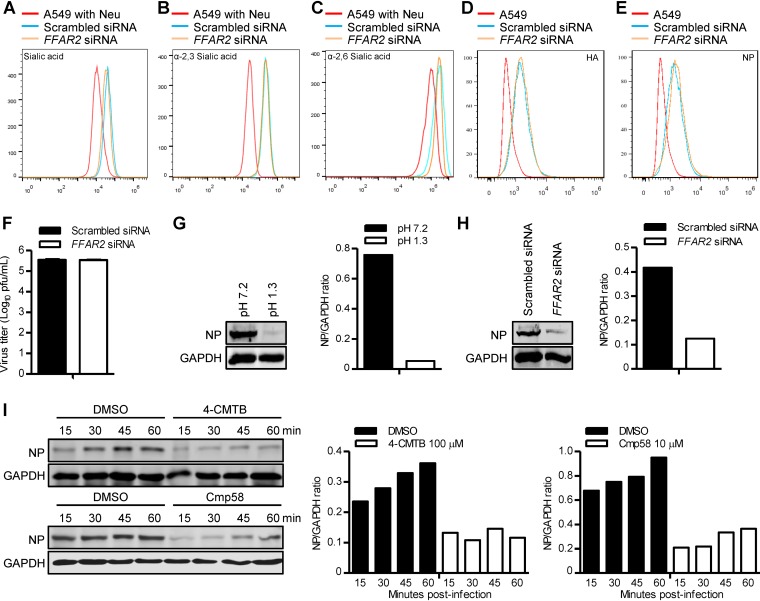
FFAR2 is important for the internalization of IAV. (A) A549 cells were treated with *FFAR2* siRNA or scrambled siRNA for 48 h, stained with a wheat germ agglutinin (WGA)-Alexa Fluor 488 conjugate, and analyzed by flow cytometry. (B and C) A549 cells treated with *FFAR2* siRNA or scrambled siRNA were stained with lectins that have specificity for α-2,3-sialic acids (MAL) (B) or α-2,6-sialic acids (SNA) (C) and analyzed by flow cytometry. (D and E) A549 cells were treated with *FFAR2* siRNA or scrambled siRNA for 48 h and then infected with AH05 (H5N1) virus on ice at 4°C for 1 h. Cells were fixed, stained with a mouse anti-HA mAb (D) or a mouse anti-NP mAb (E) and Alexa Fluor 488 goat anti-mouse IgG(H+L), and analyzed by flow cytometry. (F) A549 cells were transfected with *FFAR2* siRNA or scrambled siRNA for 48 h and infected with AH05 (H5N1) virus (MOI = 5) on ice at 4°C for 1 h. After unbound virus was washed away, the cells were collected and titrated for infectious virus by means of plaque assays on MDCK cells. (G) A549 cells were infected with AH05 (H5N1) virus (MOI = 5) on ice at 4°C for 1 h, followed by a neutral wash (ice-cold PBS, pH 7.2) or an acidic wash (ice-cold PBS-HCl, pH 1.3) before cell lysis. The amount of internalized virus particles was determined by Western blotting with a rabbit NP pAb. (H) *FFAR2* siRNA- or scrambled siRNA-treated A549 cells were infected with AH05 (H5N1) virus (MOI = 5) on ice at 4°C for 1 h, followed by a culture temperature shift to 37°C for 30 min to allow for internalization. The cells were then washed with ice-cold PBS-HCl (pH 1.3) before cell lysis. The amount of internalized virus particles was determined by Western blotting with a rabbit NP pAb. (I) A549 cells were pretreated with 100 μM 4-CMTB, 10 μM Cmp58, or DMSO for 3 h and then infected with AH05 (H5N1) (MOI = 5) at 37°C. The infected cells were washed with ice-cold PBS-HCl (pH 1.3) at the indicated time points before cell lysis. The amount of internalized virus particles was determined by Western blotting with a rabbit NP pAb. A549 cells pretreated with neuraminidase (Neu) were stained with WGA (A), MAL (B), and SNA (C), respectively, and used as negative controls. The band intensities of the Western blots were quantified by using ImageJ software and are expressed as relative NP/GAPDH ratios (G to I).

To further explore the function of FFAR2 in IAV replication, we determined the impact of FFAR2 knockdown on the early steps of the viral life cycle. Since the binding of viral HA to SA receptors on the cell surface is the first step for an IAV to invade a host cell (41), we first determined whether FFAR2 knockdown affects SA expression on the surface of the cell membrane. To this end, we used Alexa Fluor 488-conjugated wheat germ agglutinin (WGA) lectin, which specifically recognizes SA moieties, to detect whether the cell surface SAs are differentially expressed between FFAR2 knockdown A549 cells and control cells by flow cytometry analysis. FFAR2 knockdown did not affect the expression of SAs on the surface of the cell membrane (Fig. 6A). Because WGA is unable to distinguish between α-2,3- and α-2,6-SAs, we then included two additional lectins in our analysis: Maackia amurensis lectin (MAL) (specific for α-2,3-SA) and Sambucus nigra lectin (SNA) (specific for α-2,6-SA). No difference in the amounts of these two types of cell surface SA receptors was observed between FFAR2 knockdown A549 cells and control cells (Fig. 6B and C). These results demonstrate that the restricted replication of IAV in FFAR2-knocked-down cells was not caused by a change in the cell surface expression of the total or specific types of SAs.

Next, we examined the effect of siRNA-mediated knockdown of FFAR2 on viral attachment to cell membrane SA receptors. A549 cells were transfected with the *FFAR2* siRNA or control siRNA for 48 h and then infected with AH05 (H5N1) virus (MOI = 5) for 1 h on ice at 4°C, which allows virus attachment but prevents virus internalization (12). The cells were washed, trypsinized, fixed, stained with an anti-HA or an anti-NP monoclonal antibody (mAb), and then subjected to flow cytometry analysis. As shown in Fig. 6D and E, there was no difference in the percentage of membrane-bound viruses between FFAR2 knockdown and control cells, indicating that FFAR2 knockdown did not adversely affect the attachment of IAV to the cell surface. We also carried out a binding experiment with AH05 (H5N1) virus on ice at 4°C for 1 h. After washing to remove unbound viruses, the cells were frozen and thawed three times to allow for the release of viruses bound to the surface of the cells into the supernatant. The viruses in the supernatant were then titrated by the use of a plaque assay on Madin-Darby canine kidney (MDCK) cells. As shown in Fig. 6F, there was no difference in the amounts of infectious virus attached on the surface between the FFAR2 knockdown cells and the control cells. Taken together, these data indicate that FFAR2 is not required for IAV attachment to the cell surface.

The binding of viral HA protein to the SA receptors on the cell membrane rapidly initiates the endocytosis process of IAV. Therefore, we examined whether FFAR2 functions in the step of internalization after attachment of viral HA to the SA receptors. First, A549 cells were infected with AH05 (H5N1) virus (MOI = 5) for 1 h on ice at 4°C and then washed five times with ice-cold phosphate-buffered saline (PBS) (pH 7.2) or PBS-HCl (pH 1.3). As shown in Fig. 6G, the NP signal was completely lost from cells washed with ice-cold PBS-HCl (pH 1.3), whereas washing with PBS did not affect the NP signal of infected cells. Therefore, the viral NP protein was suitable to act as an internalization marker to indicate whether virions were internalized into the cells or removed from the cell surface. Next, *FFAR2* siRNA- or scrambled siRNA-treated A549 cells were infected with AH05 (H5N1) (MOI = 5) for 1 h on ice at 4°C, followed by a culture temperature shift to 37°C for 30 min to allow for internalization. The cells were then washed with ice-cold PBS-HCl (pH 1.3) to remove virions retained on the cell surface and lysed for the detection of internalized viral NP protein by Western blotting. As shown in Fig. 6H, the amount of viral NP protein inside the *FFAR2* siRNA-treated A549 cells was dramatically decreased compared with that in the control siRNA-treated cells, demonstrating that most of the IAV particles were not internalized into the infected cells when the expression of FFAR2 was downregulated. These data clearly show that FFAR2 is important for the internalization of IAV into cells.

We then investigated whether treatment with 4-CMTB or Cmp58 also has an effect on the internalization of IAV. A549 cells were pretreated with 100 μM 4-CMTB, 10 μM Cmp58, or DMSO for 3 h prior to infection with AH05 (H5N1) virus (MOI = 5) at 37°C. At 15, 30, 45, or 60 min p.i., cells were washed five times with ice-cold PBS-HCl (pH 1.3) to remove the uninternalized virions, and the cell lysates were then Western blotted. In DMSO-treated cells, the amount of internalized NP protein gradually increased over time (Fig. 6I). In contrast, only a small amount of viral NP was internalized in 4-CMTB- or Cmp58-treated A549 cells throughout the detection period from 15 to 60 min p.i. (Fig. 6I). These results indicate that the effect of 4-CMTB and Cmp58 on FFAR2 inhibits the internalization process of IAV.

**FIG 7:**
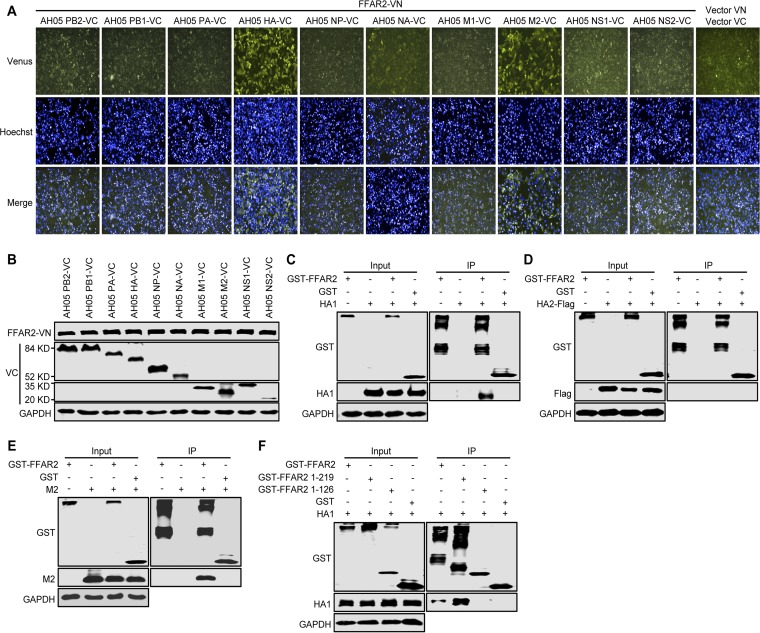
FFAR2 interacts with HA and M2 of IAV. (A) BiFC analysis to examine the interaction between FFAR2-VN- and -VC-fused proteins of AH05 (H5N1) virus in HeLa cells. Cotransfection of vector VN and vector VC served as a negative control. (B) Western blotting to detect the expression of FFAR2-VN- and -VC-fused proteins of AH05 (H5N1) virus in HeLa cells. (C to E) Co-IP assay to determine the interaction between FFAR2 and HA1, HA2, and M2 of AH05 (H5N1) virus. HEK293T cells were transfected with plasmids for the expression of GST-FFAR2 individually or in combination with HA1 (C), HA2-Flag (D), or M2 (E). Lysates of transfected cells were immunoprecipitated with glutathione magnetic agarose beads. The bound proteins were subjected to Western blotting. (F) Mapping of the FFAR2 domain that mediates the interaction with HA1. Lysates of HEK293T cells cotransfected with HA1 and FFAR2 (or its truncation mutants) were immunoprecipitated with a rabbit anti-GST pAb. The bound proteins were detected by Western blotting.

To explore the molecular mechanisms by which FFAR2 regulates IAV internalization, we attempted to detect an interaction between FFAR2 and the 10 essential IAV proteins (PB2, PB1, PA, HA, NP, NA, M1, M2, NS1, and NS2). To this end, we performed a Venus-based bimolecular fluorescence complementation (BiFC) assay in which FFAR2 was fused in frame with N-terminal residues 1 to 173 of the Venus protein (FFAR2-VN) and the AH05 (H5N1) viral proteins were fused with C-terminal residues 174 to 239 of the Venus protein (VC). The Venus fluorescence signal was detected only in HeLa cells cotransfected with FFAR2-VN/AH05 HA-VC or FFAR2-VN/AH05 M2-VC under conditions where the FFAR2-VN- and -VC-fused AH05 proteins were well expressed (Fig. 7A and B), indicating that FFAR2 interacted with only the AH05 (H5N1) HA and M2 proteins. We also performed a coimmunoprecipitation (co-IP) experiment in HEK293T cells transfected with plasmids for the expression of glutathione *S*-transferase (GST) or GST-FFAR2, together with HA1, HA2, or M2 of AH05 (H5N1) virus. HA1 and M2 specifically interacted with FFAR2 (Fig. 7C and E), whereas no interaction was observed between HA2 and FFAR2 (Fig. 7D). Given that our results prove that FFAR2 is involved in the process of AH05 (H5N1) virus internalization, the interaction between FFAR2 and HA1, the globular head domain of HA, most likely facilitates the internalization of IAV into cells. We then attempted to define the region of FFAR2 that was important for its binding with HA1. We generated two truncated FFAR2 constructs, FFAR2(1–219) and FFAR2(1–126), which were fused to the C terminus of GST and then examined for their interaction with HA1 in HEK293T cells. The domain mapping experiment indicated that the region spanning residues 1 to 219 of FFAR2 was important for the interaction of FFAR2 with AH05 (H5N1) HA1 (Fig. 7F).

Taken together, these results demonstrate that FFAR2 is an important cofactor for the internalization of IAV.

### The AP-2 complex, in coordination with β-arrestin1, is involved in FFAR2-mediated IAV endocytosis.

**FIG 8:**
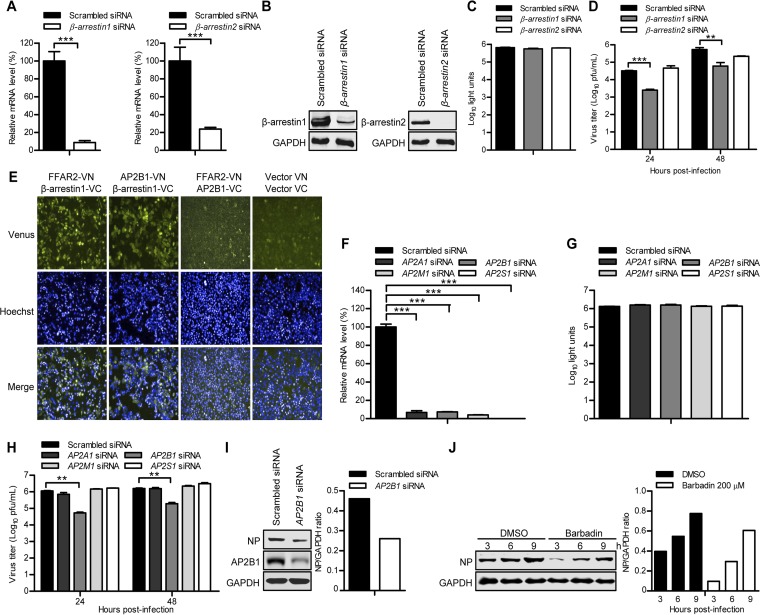
The FFAR2 downstream effectors β-arrestin1 and AP2B1 promote the replication of IAV. (A) A549 cells were transfected with siRNA targeting β-*arrestin1* or β-*arrestin2* or with scrambled siRNA for 48 h. The levels of β-*arrestin1* or β-*arrestin2* mRNA in β-*arrestin1* or β-*arrestin2* siRNA-treated cells were determined by qRT-PCR in cell lysates and standardized to those in the scrambled siRNA-treated cells. ***, *P < *0.001. (B) β-Arrestin1- or β-arrestin2-overexpressing cells that were generated by using retrovirally mediated transduction were transfected with siRNA targeting β-*arrestin1* or β-*arrestin2* or with scrambled siRNA for 48 h. Whole-cell lysates were collected and subjected to Western blotting with a mouse anti-β-arrestin1 mAb or a mouse anti-β-arrestin2 mAb. (C) Cell viability of β-*arrestin1* siRNA- or β-*arrestin2* siRNA-treated A549 cells was measured by using the CellTiter-Glo assay. (D) A549 cells were transfected with siRNA targeting β-*arrestin1* or β-*arrestin2* or with scrambled siRNA for 48 h and then infected with AH05 (H5N1) virus (MOI = 0.1). Supernatants were collected at the indicated time points, and virus titers were determined by means of plaque assays on MDCK cells. **, *P < *0.01; ***, *P < *0.001. (E) BiFC analysis to determine the interaction between FFAR2-VN and β-arrestin1-VC, AP2B1-VN and β-arrestin1-VC, and FFAR2-VN and AP2B1-VC in HeLa cells. Cotransfection of vector VN and vector VC served as a negative control. (F) A549 cells were transfected with siRNAs targeting different subunits of the AP-2 protein complex for 48 h. The levels of mRNA of the indicated gene in the cell lysates were determined by qRT-PCR and standardized to those in the scrambled siRNA-treated cells. ***, *P < *0.001. (G) A549 cells were treated with siRNAs targeting different subunits of the AP-2 protein complex for 48 h, and cell viability was measured by using the CellTiter-Glo assay. (H) A549 cells were transfected with siRNA targeting the indicated gene of the AP-2 protein complex or with scrambled siRNA for 48 h and then infected with AH05 (H5N1) virus (MOI = 0.1). Supernatants were collected at 24 and 48 h p.i., and virus titers were determined by means of plaque assays on MDCK cells. **, *P < *0.01. (I) *AP2B1* siRNA- or scrambled siRNA-treated A549 cells were infected with AH05 (H5N1) virus (MOI = 5) on ice at 4°C for 1 h, followed by a culture temperature shift to 37°C for 30 min to allow for internalization. The cells were then washed with ice-cold PBS-HCl (pH 1.3) before cell lysis. The amount of internalized virus particles was determined by Western blotting with a rabbit anti-NP pAb. (J) A549 cells were pretreated with 200 μM Barbadin or DMSO for 12 h and then infected with AH05 (H5N1) virus (MOI = 5). Whole-cell lysates were collected at the indicated time points and subjected to Western blotting with a rabbit anti-NP pAb. The band intensities of the Western blots were quantified by using ImageJ software and are expressed as relative NP/GAPDH ratios (I and J).

Studies have established that β-arrestins are essential for the internalization of many GPCRs (42), acting as adaptors that link the receptors to clathrin-coated pits (43). Mammalian cells express four arrestin members, arrestin-1, β-arrestin1, β-arrestin2, and arrestin-4, of which only the β-arrestins (β-arrestin1 and β-arrestin2) are ubiquitously expressed (44). We therefore asked whether β-arrestins were recruited during FFAR2-mediated IAV endocytosis by evaluating the effect of β-arrestin1 and β-arrestin2 downregulation on IAV infection. Reverse transcription-quantitative PCR (qRT-PCR) and Western blotting showed that the expression levels of β-arrestin1 and β-arrestin2 were reduced in specific siRNA-treated A549 cells and retrovirally mediated β-arrestin1- or β-arrestin2-overexpressing cells but not in cells treated with nontargeting siRNA (Fig. 8A and B). siRNA knockdown of β-arrestin1 and β-arrestin2 had no toxic effects on the viability of A549 cells (Fig. 8C). The siRNA-treated A549 cells were infected with AH05 (H5N1) virus (MOI = 0.1), and the virus titers in the supernatant were determined at 24 and 48 h p.i. As shown in Fig. 8D, only β-arrestin1 downregulation led to a significant decrease in virus titers compared with the scrambled siRNA-treated cells. We then investigated whether β-arrestin1 was involved in FFAR2-mediated IAV endocytosis. To achieve this, we conducted a BiFC assay in which FFAR2 was fused in frame with N-terminal residues 1 to 173 of Venus protein (FFAR2-VN) and β-arrestin1 was fused with C-terminal residues 174 to 239 of Venus (β-arrestin1-VC). We detected an intense Venus fluorescence signal in HeLa cells cotransfected with the two fusion constructs, indicating that β-arrestin1 strongly interacts with FFAR2 (Fig. 8E).

It has been reported that β-arrestin1 directly interacts with the β2-subunit of the AP-2 complex (45), which is a critical component of the CME pathway. We therefore investigated whether the AP-2 complex was also involved in FFAR2-mediated IAV endocytosis. The effect of the AP-2 subunits AP2A1, AP2B1, AP2M1, and AP2S1 on IAV infection was then determined by means of siRNA silencing. The expression of AP2A1, AP2B1, AP2M1, and AP2S1 was reduced in specific siRNA-treated A549 cells without causing cytotoxic effects (Fig. 8F and G). The gene-specific siRNA-treated A549 cells were infected with AH05 (H5N1) virus (MOI = 0.1), and the virus titers in the supernatant were determined at 24 and 48 h p.i. As shown in Fig. 8H, only AP2B1 downregulation significantly decreased the virus titers compared with the control siRNA-treated cells. Next, *AP2B1* siRNA- or scrambled siRNA-treated A549 cells were infected with AH05 (H5N1) (MOI = 5) for 1 h on ice at 4°C, shifted to 37°C for 30 min to allow for internalization, washed with ice-cold PBS-HCl (pH 1.3) to remove virions retained on the cell surface, and lysed for the detection of internalized viral NP by Western blotting. As shown in Fig. 8I, the amount of viral NP protein inside the *AP2B1* siRNA-treated A549 cells was dramatically decreased compared with that in the control siRNA-treated cells, demonstrating that most of the IAV particles were not internalized into the infected cells when the expression of AP2B1 was downregulated. BiFC analysis, by cotransfecting HeLa cells with FFAR2-VN with AP2B1-VC, produced almost no Venus fluorescence; instead, a strong Venus fluorescence signal was observed in HeLa cells cotransfected with AP2B1-VN and β-arrestin1-VC (Fig. 8E).To further investigate the role of AP2B1, through its interaction with β-arrestin1, in the FFAR2-mediated internalization of IAV, we used a specific compound, Barbadin, to selectively inhibit the interaction between AP2B1 and β-arrestin1 (46). A549 cells were pretreated with Barbadin (200 μM) for 12 h and then infected with AH05 (H5N1) (MOI = 5). We found that the amount of NP protein internalized in Barbadin-treated A549 cells was reduced at 3, 6, and 9 h compared with that in DMSO-treated A549 cells (Fig. 8J). These results suggest that the AP-2 complex is involved in FFAR2-mediated IAV endocytosis not by directly interacting with FFAR2 but by relying on the bridging role of β-arrestin1.

### G protein-coupled receptor kinases are required for FFAR2-mediated IAV infection.

**FIG 9:**
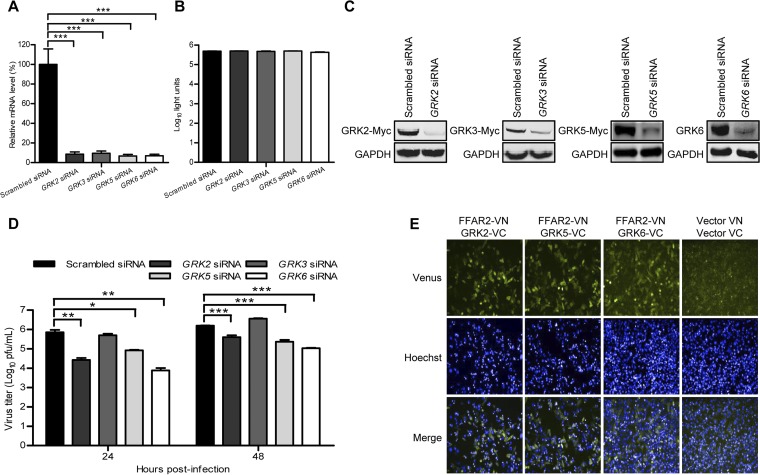
G protein-coupled receptor kinases (GRKs) are required for IAV replication. (A) A549 cells were transfected with siRNA targeting *GRK2*, *GRK3*, *GRK5*, or *GRK6* for 48 h. The levels of mRNA of the indicated gene in the cell lysates were determined by qRT-PCR and standardized to those of the scrambled siRNA-treated cells. ***, *P < *0.001. (B) A549 cells were treated with siRNA targeting *GRK2*, *GRK3*, *GRK5*, or *GRK6* for 48 h, and cell viability was measured by using the CellTiter-Glo assay. (C) HEK293T cells were treated with siRNA targeting *GRK2*, *GRK3*, or *GRK5* or with scrambled siRNA for 12 h and then transfected with the corresponding Myc-tagged protein expression construct for 36 h. Whole-cell lysates were collected and analyzed by Western blotting with a mouse anti-Myc mAb. Separately, A549 cells were transfected with *GRK6* siRNA or scrambled siRNA for 48 h, and whole-cell lysates were collected and analyzed by Western blotting with a rabbit anti-GRK6 pAb. (D) A549 cells were transfected with siRNA targeting *GRK2*, *GRK3*, *GRK5* or *GRK6* or with scrambled siRNA for 48 h and then infected with AH05 (H5N1) virus (MOI = 0.1). Supernatants were collected at the indicated time points, and virus titers were determined by means of plaque assays on MDCK cells. *, *P < *0.05; **, *P < *0.01; ***, *P < *0.001. (E) BiFC analysis to examine the interaction between FFAR2-VN and GRK2-VC, GRK5-VC, or GRK6-VC in HeLa cells. Cotransfection of vector VN and vector VC served as a negative control.

Most of the GPCRs are phosphorylated at the C terminus by GRKs (47), followed by binding of β-arrestins to mediate the internalization of the receptor and the subsequent signaling cascade (48). There are seven isoforms of GRKs, GRK1 to GRK7, among which only GRK2, GRK3, GRK5, and GRK6 are widely expressed in different cell types (49). We therefore assessed the role of GRK2, GRK3, GRK5, and GRK6 in the regulation of IAV replication by means of siRNA silencing. Real-time PCR showed that the expression of GRK2, GRK3, GRK5, or GRK6 was reduced in specific siRNA-treated A549 cells but not in cells treated with control siRNA (Fig. 9A) and that siRNA treatment had no toxic effect on A549 cell viability (Fig. 9B). The knockdown efficiency of siRNAs was also validated by the reduced expression of cotransfected GRK2, GRK3, and GRK5 protein expression constructs in HEK293T cells as well as by the decreased endogenous expression of GRK6 protein in A549 cells (Fig. 9C). The siRNA-treated A549 cells were infected with AH05 (H5N1) virus (MOI = 0.1), and the virus titers in the supernatant were determined at 24 and 48 h p.i. As shown in Fig. 9D, downregulation of three of the four GRKs, that is, GRK2, GRK5, and GRK6, dramatically decreased the virus titers compared with those in the scrambled siRNA-treated cells. We then investigated whether GRK2, GRK5, or GRK6 interacts with FFAR2. HeLa cells were cotransfected with FFAR2-VN and GRK2-VC, GRK5-VC, or GRK6-VC, in which the GRK2, GRK5, or GRK6 protein was fused with C-terminal residues 174 to 239 of Venus protein, respectively. As shown in Fig. 9E, a strong Venus fluorescence signal was detected in HeLa cells cotransfected with FFAR2-VN and GRK2-VC, GRK5-VC, or GRK6-VC, which indicates that GRK2, GRK5, and GRK6 interact with FFAR2. Together, these results suggest that GRK2-, GRK5-, or GRK6-mediated phosphorylation of FFAR2 facilitates downstream signaling by promoting the recruitment of β-arrestin1 and AP2B1, ultimately enhancing the endocytosis of IAV through the CME pathway.

## DISCUSSION

The initial step of IAV infection is to bind to SA receptors conjugated to glycoproteins and glycolipids on the cell surface. This binding triggers subsequent viral entry steps. For IAV, the principal route of entry into host cells is the CME pathway. A number of studies have demonstrated that IAV internalization requires the engagement of various cellular factors, for example, the EPN1 adaptor protein (11), and the involvement of different signaling pathways, such as the recruitment of receptor tyrosine kinases (EGFR and MET) (13) and the subsequent activation of PI3K (14), PLC-γ1 (15), or protein kinase C (PKC) (50). However, the role of these factors was mostly demonstrated in a limited number of IAV subtypes, such as H1N1 or H3N2. Moreover, it has been reported that distinct signaling pathways are specifically activated by different IAV subtypes to facilitate virus internalization (15, 51). These facts prompted us to investigate potential cofactors involved in the entry of H5N1 virus into host cells.

In the present study, we identified the host cellular protein FFAR2, a seven-transmembrane GPCR receptor, as an important cofactor for the replication of IAV, especially the H5N1 subtype, by using a genome-wide RNA interference (RNAi) screen. Knockdown of FFAR2 by using siRNA treatment can universally restrict the replication of different subtypes of IAV, and the inhibitory effect of *FFAR2* knockout on IAV replication can be rescued by retrovirally mediated FFAR2 complementation in *FFAR2*_KO A549 cells. We found that the effect of siRNA knockdown of FFAR2 expression on IAV replication does not involve the stimulation of the IFN pathway because the level of Mx1 protein was unchanged and ISRE reporter activity was unstimulated. Furthermore, treatment of A549 cells with *FFAR2* siRNA or the FFAR2 agonists 4-CMTB and Cmp58 significantly inhibited both the nuclear accumulation and cytoplasm localization of vRNP complexes at early time points after IAV infection but had no effect on viral genome transcription or replication. These results demonstrate that FFAR2 is involved in an early stage of the viral replication cycle prior to the transcription and replication of the viral genome. In an effort to define the specific stage of IAV replication that involves FFAR2, we first showed that FFAR2 knockdown in A549 cells had no effect on the cell surface expression of SA receptors and that the attachment of IAV particles to the SA receptors was also not affected. However, the attached viruses on the surface of cells treated with *FFAR2* siRNA, 4-CMTB, or Cmp58 were easily removed by washes with PBS-HCl (pH 1.3), resulting in a significant reduction in the levels of viral NP internalized in the infected cells. Of note, the treatment of A549 cells with 4-CMTB or Cmp58 inhibited the replication of H5N1 virus in A549 cells. Mechanistically, sustained stimulation with agonists could cause internalization of FFAR2, resulting in a decrease in the amount of FFAR2 on the cell surface. In this case, insufficient FFAR2 on the cell membrane would be unable to efficiently facilitate the internalization of IAV through the endocytosis pathway. By using the BiFC assay and the co-IP experiment, we demonstrate that FFAR2 interacted with viral HA and M2 among the 10 essential proteins of IAV. We speculate that the interaction between FFAR2 and HA most likely stimulates the FFAR2 signaling cascade, promoting IAV internalization after HA attachment to the SA receptors on the cell surface. Compared with HA, the amount of M2 on viral particles is small. Moreover, M2 mainly functions at the uncoating stage during IAV entry; i.e., it serves as an ion channel to acidify the interior of the virus particle, resulting in vRNP complex detachment from M1 and release into the cytoplasm. We demonstrate that FFAR2 is important in the internalization step during IAV entry. However, whether FFAR2 is also involved in the uncoating step by interacting with M2 requires further investigation. Collectively, these findings demonstrate that FFAR2 is an important cofactor for IAV entry into host cells.

The mechanism of FFAR2 function during IAV uptake was unraveled by dissecting the roles of associated adaptors in the signaling cascade. Typically, mammalian GPCR endocytosis upon activation is dependent primarily on the regulation of arrestins through a CME pathway (52). GRKs are involved in this process and specifically phosphorylate activated GPCRs (53). In our study, we found that GRK2, GRK5, and GRK6 knockdown in A549 cells inhibited IAV replication. However, it remains unknown whether these kinases simultaneously or sequentially regulate the phosphorylation of FFAR2. In addition, Western blotting showed that only GRK6 in A549 cells was abundantly expressed, and the endogenous expression levels of GRK2 and GRK5 were relatively low. Taking into account that GRK6 knockdown produced the most significant inhibitory effect on IAV replication in A549 cells, we speculated that GRK6 may play a more important role in the C-terminal phosphorylation of FFAR2 during IAV infection than GRK2 and GRK5.

The arrestins are a family of four proteins: arrestins 1 and 4 are primarily expressed in the eye and are associated with visual transduction, whereas the nonvisual arrestins 2 and 3 (also known as β-arrestin1 and β-arrestin2) are more widely expressed (54). The ubiquitously expressed β-arrestin1 and β-arrestin2 are best known for functioning in the regulation of phosphorylated GPCR desensitization and internalization (52). To date, β-arrestin1 and β-arrestin2 have been reported to be involved in the internalization process of many viruses, such as HIV (55) and JC polyomavirus (56). The GPCR family proteins have different binding affinities for β-arrestin1 and β-arrestin2 (53). Lee et al. used a bimolecular luminescence complementation assay and coimmunoprecipitation to demonstrate that FFAR2 can interact with β-arrestin1 and β-arrestin2 (57). In our study, the introduction of β-*arrestin1* siRNA into A549 cells attenuated H5N1 virus replication, whereas treatment with β-*arrestin2* siRNA had no observable effect. It may be that β-arrestin1 interacts with FFAR2 with a higher affinity than β-arrestin2 during H5N1 virus infection.

The AP-2 adaptor complex is a heterotetramer consisting of α (AP2A1), β2 (AP2B1), μ2 (AP2M1), and σ2 (AP2S1) adaptin subunits (52, 58). It plays multiple roles in CME, such as promoting the assembly of clathrin-coated pits and coordinating interactions between clathrin and other CME proteins (59, 60). To date, the AP-2 complex has been reported to be involved in the endocytosis processes of different viruses, including HIV (61, 62), murine cytomegalovirus (63), and white spot syndrome virus (64). As for IAV, a previous study by Chen and Zhuang reported that siRNA knockdown of the AP2M1 subunit of the AP-2 complex did not inhibit the clathrin-mediated uptake of IAV (11). Here, we also found that AP2M1 knockdown had no effect on the replication of H5N1 virus. Interestingly, among the four subunits of the AP-2 complex, only AP2B1 knockdown significantly inhibited the replication of H5N1 virus. Although AP2B1 did not interact with FFAR2, according to our BiFC assay, it clearly interacted with β-arrestin1, which is consistent with a previous report that β-arrestins target the GPCRs to clathrin-coated vesicle-mediated endocytosis via their direct interaction with the AP2B1 subunit of the AP-2 complex (45). Moreover, Barbadin, by selectively targeting the interaction between β-arrestin1 and AP2B1, also inhibited the internalization of IAV. Together, these results suggest that the AP2B1 subunit of the AP-2 complex acts synergistically with clathrin and β-arrestin1 in the FFAR2-mediated endocytosis of IAV.

**FIG 10:**
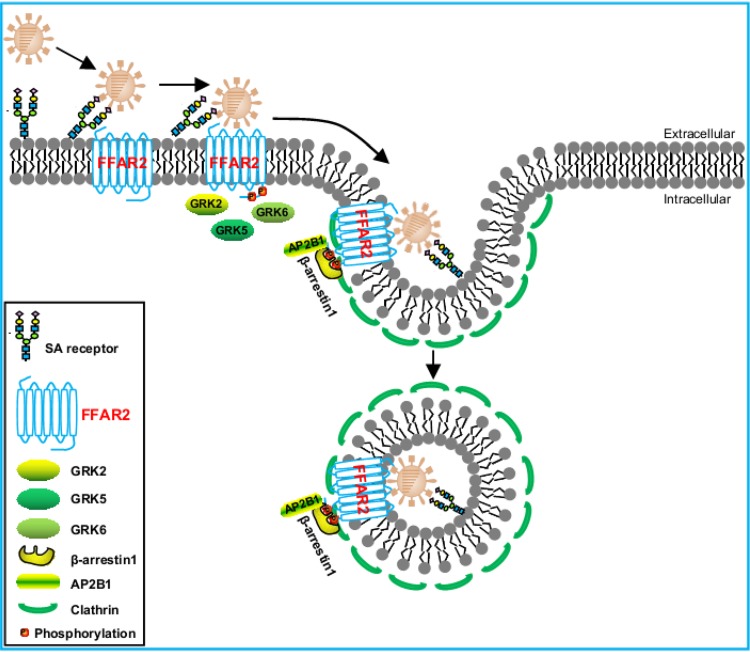
Model of the role of FFAR2 in IAV entry into host cells. Upon initial binding with sialic acid on the cell surface, IAV binds to the FFAR2 protein, the C terminus of which is phosphorylated by the GRKs (i.e., GRK2, GRK5, or GRK6). β-Arrestin1 then translocates to the plasma membrane and interacts with FFAR2, followed by further interaction with AP2B1. Ultimately, the FFAR2–β-arrestin1–AP2B1 complex aids in the internalization of IAV via clathrin-mediated endocytosis.

In summary, here, we demonstrate that the G protein-coupled receptor FFAR2 is required for the efficient replication of IAV. FFAR2 plays a key role in the step of IAV entry into host cells. We further reveal that the C-terminal phosphorylation of FFAR2 by GRK2, GRK5, or GRK6 is important for FFAR2 to support virus replication. Moreover, we found that the FFAR2–β-arrestin1–AP2B1 signaling cascade is necessary for the efficient endocytosis of IAV into host cells (Fig. 10). Together, our data suggest that FFAR2 is a crucial host factor for influenza virus entry into host cells.

## MATERIALS AND METHODS

### Cells and viruses.

Human embryonic kidney cells (HEK293T), human lung carcinoma cells (A549), Madin-Darby canine kidney (MDCK) cells, and murine macrophage cells (RAW 264.7) were cultured at 37°C in a 5% CO_2_ humidified incubator in Dulbecco’s modified Eagle’s medium (DMEM; Life Technologies, Grand Island, NY) supplemented with 10% fetal bovine serum (FBS; Sigma-Aldrich, St. Louis, MO), F-12K medium (Life Technologies) supplemented with 10% FBS, DMEM supplemented with 6% newborn calf serum (NCS; Sigma-Aldrich), and RPMI 1640 medium (Life Technologies) containing 10% FBS, respectively. All media contained 100 U/ml penicillin and 100 μg/ml streptomycin (Life Technologies).

Venus-expressing reporter influenza viruses, H1N1 NA-Venus, H5N1 NA-Venus, and H9N2 NA-Venus, were generated in our laboratory as described previously (38). All experiments with wild-type A/Anhui/2/2005 (AH05) (H5N1) virus or with H5N1 NA-Venus reporter virus were conducted within the enhanced animal biosafety level 3 (ABSL3^+^) facility in the Harbin Veterinary Research Institute (HVRI) of the Chinese Academy of Agricultural Sciences (CAAS), which is approved for such use by the Ministry of Agriculture and Rural Affairs of China and the China National Accreditation Service for Conformity Assessment.

### Plasmids.

The *FFAR2*, *GRK3*, *GRK5*, *GRK6*, β-*arrestin1*, and β-*arrestin2* genes were amplified by RT-PCR from total cellular mRNAs of A549 cells. The *GRK2* gene was synthesized at Comate Bioscience Co., Ltd. (Changchun, China), due to its low abundance in A549 cells. *GRK2*, *GRK3*, and *GRK5* were subsequently cloned into the mammalian expression vector pCAGGS, which contains a Myc tag at the C terminus. pRetroX-IRES-ZsGreen1-β-arrestin1 and pRetroX-IRES-ZsGreen1-β-arrestin2 were constructed by inserting the open reading frame (ORF) of β-*arrestin1* or β-*arrestin2* into the pLVX-IRES-ZsGreen1 vector (Clontech, Mountain View, CA). pQCXIN-FFAR2 was constructed by inserting the *FFAR2* ORF into the pQCXIN vector (Clontech). GST-tagged FFAR2, FFAR2(1–219), and FFAR2(1–126) were constructed in pCAGGS with a GST tag at the N terminus. The ORFs of *FFAR2* and *AP2B1* were fused with the Venus N terminus (VN) (residues 1 to 173), and the ORFs of *AP2B1*, β-*arrestin1*, *GRK2*, *GRK5*, *GRK6*, and 10 essential proteins of AH05 (H5N1) virus were fused with the Venus C terminus (VC) (residues 174 to 239), which were then cloned into pCAGGS. *HA1* and *M2* of AH05 (H5N1) virus were cloned into pCAGGS, and *HA2* was cloned into pCAGGS with a Flag tag at the C terminus. The primer sequences of all oligonucleotides used for cloning are available upon request.

### Antibodies and compounds.

The commercially obtained primary antibodies used in this study were as follows: mouse anti-Flag monoclonal antibody (mAb) (catalog number F3165; Sigma-Aldrich), mouse anti-GST mAb (catalog number A00865; GenScript, Nanjing, China), mouse anti-HA1 mAb (catalog number ab135382; Abcam, Cambridge, MA), mouse anti-Myc mAb (catalog number M4439; Sigma-Aldrich), mouse anti-β-arrestin1 mAb (catalog number sc-74591; Santa Cruz, Dallas, TX), mouse anti-β-arrestin2 mAb (catalog number sc-13140; Santa Cruz), rabbit anti-AP2B1 polyclonal antibody (pAb) (catalog number 15690-1-AP; Proteintech, Wuhan, China), rabbit anti-FFAR2 pAb (catalog number ABC299; Sigma-Aldrich), rabbit anti-GAPDH pAb (catalog number 10494-1-AP; Proteintech), rabbit anti-GRK6 pAb (catalog number GTX54160; GeneTex, Irvine, CA), rabbit anti-GST pAb (catalog number A00097; GenScript), rabbit anti-LaminB1 pAb (catalog number 12987-1-AP; Proteintech), and rabbit anti-Mx1 pAb (catalog number 13750-1-AP; Proteintech). The mouse anti-NP mAb and rabbit anti-NP pAb were made and stored in our laboratory. Alexa Fluor 488 goat anti-mouse IgG(H+L) (catalog number A11029) and Alexa Fluor 633 goat anti-rabbit IgG(H+L) (catalog number A21071), obtained from Life Technologies, were used as secondary antibodies for confocal microscopy. The secondary antibodies used for Western blotting were DyLight 800 goat anti-mouse IgG(H+L) (catalog number 072-07-18-06) and DyLight 800 goat anti-rabbit IgG(H+L) (catalog number 072-07-15-06), purchased from KPL (Gaithersburg, MD). Agonists of FFAR2, 4-CMTB (catalog number SML0302) and Cmp58 [(*S*)-2-(4-chlorophenyl)-3,3-dimethyl-*N*-(5-phenylthiazol-2-yl)butanamide] (catalog number 371725), were purchased from Sigma-Aldrich. An inhibitor of the β-arrestin1/AP2B1 complex, Barbadin {3-amino-5-[4-(phenylmethyl)phenyl]thieno[2,3-d]pyrimidin-4(3H)-one} (catalog number B118250), was purchased from TRC (Ontario, Canada).

### Effect of siRNA knockdown of FFAR2 on the replication of reporter IAVs.

A549 cells were seeded into 96-well plates and then transfected with *FFAR2* siRNA (5′-GCCUCUGUAUGGAGUGAUU-3′) or with a scrambled siRNA (GenePharma, Shanghai, China) at a concentration of 30 nM by using the Lipofectamine RNAiMAX transfection reagent (Invitrogen, Carlsbad, CA). At 48 h posttransfection, knockdown of FFAR2 was verified by Western blotting with a rabbit anti-FFAR2 pAb. siRNA-treated A549 cells were infected for 24 h with H1N1 NA-Venus, H5N1 NA-Venus, or H9N2 NA-Venus virus (MOI = 0.1). Cells were then fixed with 4% paraformaldehyde (PFA) and stained for nuclei by using Hoechst 33342. Images were captured by using the Operetta high-content imaging system (PerkinElmer, Waltham, MA) and used to calculate the cell infection ratio according to the Venus fluorescence intensity. The inhibitory effect of siRNA on virus replication is shown as a relative ratio, which was calculated by dividing the average fluorescence intensity of three cell wells containing *FFAR2* siRNA with that of three wells containing scrambled siRNA. In an independent experiment, A549 cells that were treated with siRNA and infected as described above were fixed with 4% PFA, permeabilized with 0.5% Triton X-100 in PBS for 15 min, and incubated with blocking buffer containing 5% bovine serum albumin (BSA) for 1 h at room temperature. The viral NP protein was stained by immunostaining with a rabbit anti-NP pAb and goat anti-rabbit IgG(H+L) conjugated with Alexa Fluor 633. The nuclei were stained with Hoechst 33342. Blue and red fluorescence images were collected from the same area and used to quantify the percentage of infected cells. The percentage of infection was calculated as follows: number of cells expressing NP protein (red)/total number of cells (blue) (>300 cells were examined).

### Effect of siRNA knockdown of FFAR2 and its downstream genes on the replication of IAV or VSV-EGFP.

siRNA targeting *FFAR2*, β-*arrestin1* (5′-AAAGCCUUCUGCGCGGAGAAU-3′), β-*arrestin2* (5′-GGCUCAACUCGAACAAGAU-3′), *GRK2* (5′-GCCCAUUCAUUGUCUGCAU-3′), *GRK3* (5′-GCACUACCACCUUUCACAA-3′), *GRK5* (5′-GAAGGACCAUAGACAGAGA-3′), *GRK6* (5′-GCGGAUCAAGAAGCGGAAA-3′), *AP2A1* (5′-GCCGCAUGUAUCUCUUCUA-3′), *AP2B1* (5′-CCAUGAUAUCAAUGCCCAA-3′), *AP2M1* (5′-GUGGAUGCCUUUCGGGUCA-3′), or *AP2S1* (5′-GACCUGGUGUUCAACUUCU-3′) or scrambled siRNA at a concentration of 30 nM was transfected into A549 cells seeded into 12-well plates by using the Lipofectamine RNAiMAX transfection reagent. At 48 h posttransfection, the knockdown efficiency was determined by qRT-PCR or Western blotting. The siRNA-treated A549 cells were infected with WSN (H1N1) (MOI = 0.01), AH05 (H5N1) (MOI = 0.1), or SH13 (H9N2) (MOI = 0.1) virus. Supernatants were collected at the indicated time points, and virus titers were determined by means of plaque assays on MDCK cells (65).

siRNA targeting *FFAR2* or scrambled siRNA at a concentration of 30 nM was transfected into A549 cells seeded into 12-well plates by using the Lipofectamine RNAiMAX transfection reagent. At 48 h posttransfection, the siRNA-treated A549 cells were infected with VSV-EGFP (100 50% tissue culture infective doses [TCID_50_]). Supernatants were collected at the indicated time points and titrated on HEK293T cells by calculating the TCID_50_.

siRNA targeting *Ffar2* (5′-GCCACUGUAUGGAGUGAUC-3′) or scrambled siRNA at a concentration of 30 nM was transfected into mouse RAW 264.7 cells seeded into 12-well plates by using the Lipofectamine RNAiMAX transfection reagent. At 48 h posttransfection, the knockdown efficiency was determined by Western blotting with a rabbit anti-FFAR2 pAb. The siRNA-treated mouse RAW 264.7 cells were infected with AH05 (H5N1) virus (MOI = 0.1 or 5). Supernatants were collected at 24 and 48 h p.i. (MOI = 0.1) or at 8 h p.i. (MOI = 5), and virus titers were determined by means of plaque assays on MDCK cells.

### Generation of *FFAR2*_KO A549 cells and virus infection.

*FFAR2*_KO A549 cells were established using the CRISPR/Cas9 system. Each of the four sgRNA sequences (5′-CGCAGCCTCGATGATCTTGA-3′, 5′-GTCTGCGCCCTCACGAGTTT-3′, 5′-CACCGATAACCAGTTGGACG-3′, and 5′-CTGGTGGCGGTCAATAGCCG-3′) was inserted into the pSpCas9(BB)-2A-GFP (pX458) vector (66), which also contains an expression cassette for Cas9 and EGFP. Two micrograms of each pX458 plasmid containing one of the *FFAR2*-targeting sequences was simultaneously electrotransfected into A549 cells by using the Neon transfection system (Thermo Fisher Scientific, Waltham, MA). The electrotransfected cells were trypsinized 48 h later, and single cells were then seeded into each well of a 96-well plate by using fluorescence-activated cell sorting (FACS) with a MoFlo XDP cell sorter (Beckman Coulter, Brea, CA). The knockout of FFAR2 expression was confirmed by PCR, sequencing, and Western blotting. The *FFAR2*_KO A549 cells or control cells were infected with AH05 (H5N1) virus at an MOI of 0.1. Supernatants were collected at 24 and 48 h p.i., and virus titers were determined by means of plaque assays on MDCK cells.

### Complementation of FFAR2 in *FFAR2*_KO A549 cells and virus infection.

The retroviral construct pQCXIN-FFAR2 or the empty pQCXIN vector was transfected into the AmphoPack-293 packaging cell line (catalog number 631505; Clontech) cultured in 10-cm dishes by using Lipofectamine LTX and Plus reagents. At 48 h posttransfection, viral supernatants from the packaging cell line were collected and used to transduce *FFAR2*_KO A549 cells cultured in 6-well plates. Twenty-four hours later, *FFAR2*_KO A549 cells were retransduced with viral supernatants. The confluent transduced cells were trypsinized and cultured for 1 week in medium supplemented with 1,000 μg/ml G418 for selection. The surviving cells were individually cloned in 96-well plates by using a MoFlo XDP cell sorter (Beckman Coulter) and propagated, resulting in the generation of *FFAR2*_KO_Comp A549 cells. The complementation of FFAR2 in *FFAR2*_KO_Comp A549 cells was verified by Western blotting. The *FFAR2*_KO_Comp A549 cells or control A549 cells were infected with AH05 (H5N1) (MOI = 0.1) virus. Supernatants were collected at 24 and 48 h p.i., and virus titers were determined by means of plaque assays on MDCK cells.

### Generation of β-arrestin1- and β-arrestin2-overexpressing cells and siRNA knockdown efficiency.

pRetroX-IRES-ZsGreen1-β-arrestin1 or pRetroX-IRES-ZsGreen1-β-arrestin2 was cotransfected with psPAX2 and pMD2.G into HEK293T cells by using Lipofectamine LTX and Plus reagents (Invitrogen). At 48 h posttransfection, viral supernatants were collected and used to transduce A549 cells cultured in 6-well plates. Twenty-four hours later, A549 cells were retransduced with viral supernatants to enrich for transductants. After a 24-h incubation, green fluorescent protein (GFP)-positive cells were harvested by using a MoFlo XDP cell sorter (Beckman Coulter) and propagated in 6-well plates. The overexpression of β-arrestin1 or β-arrestin2 was tested by Western blotting with corresponding antibodies. To determine the knockdown efficiency of siRNA targeting β-*arrestin1* or β-*arrestin2*, the β-arrestin1- or β-arrestin2-overexpressing cells were transfected with siRNA targeting β-*arrestin1* or β-*arrestin2* or with scrambled siRNA at a concentration of 30 nM for 48 h by using the Lipofectamine RNAiMAX transfection reagent. The siRNA knockdown efficiency was checked by Western blotting.

### qRT-PCR.

Total RNA was extracted by using the RNeasy Plus minikit (Qiagen, Valencia, CA) according to the manufacturer’s instructions. The first-strand cDNAs were synthesized with an oligo(dT) primer and random 6-mers by using the PrimeScript RT reagent kit with gDNA Eraser (TaKaRa, Dalian, China). qRT-PCR assays were performed by using SYBR Premix Ex *Taq* II (Tli RNase H Plus; TaKaRa). Relative RNA quantities were determined by using the comparative cycle threshold method, with the cellular GAPDH gene serving as the internal control. Dissociation curve analysis was performed after each assay to ensure specific detection. The qRT-PCR primer sequences are available upon request.

### Cell viability assay.

Cell viability was determined by using the CellTiter-Glo kit (Promega, Madison, WI) as described previously (65, 67). Briefly, A549 cells seeded in opaque-walled 96-well plates containing 100 or 10 μM 4-CMTB or Cmp58 were cultured for 24 h, and A549 cells transfected with different siRNAs at a concentration of 30 nM were cultured for 48 h. CellTiter-Glo reagent (100 μl) was then added directly into each well to induce cell lysis on a shaker for 10 min before luminescence was measured with a GloMax 96 microplate luminometer (Promega).

### Effect of FFAR2 knockdown or treatment with 4-CMTB or Barbadin on the expression of IAV NP.

A549 cells grown in 12-well plates were transfected with *FFAR2* siRNA (30 nM) or scrambled siRNA or were treated with 4-CMTB (100 μM), Barbadin (200 μM), or DMSO, followed by infection with AH05 (H5N1) virus at an MOI of 5. At 3, 6, or 9 h p.i., cell lysates were subjected to Western blotting with a rabbit anti-NP pAb to determine the level of NP protein expression.

### Nuclear and cytoplasmic fractionation.

*FFAR2* siRNA- or scrambled siRNA-treated A549 cells grown in 6-cm dishes were infected with AH05 (H5N1) virus at an MOI of 5. At 2 and 3 h p.i., the cells were separated into nuclear and cytoplasmic fractions by using NE-PER nuclear and cytoplasmic extraction reagents (Pierce, Rockford, IL) according to the manufacturer’s protocol. Briefly, the siRNA-treated cells were trypsinized and centrifuged at 2,000 rpm for 2 min. The cell pellet was suspended in 200 μl of cytoplasmic extraction reagent I containing phenylmethylsulfonyl fluoride (PMSF) (Beyotime, Shanghai, China) and a protease cocktail inhibitor (Roche Diagnostics GmbH, Mannheim, Germany). The suspension was incubated on ice for 10 min before 11 μl of ice-cold cytoplasmic extraction reagent II was added; the suspension was then vortexed for 5 s, incubated on ice for 1 min, and then centrifuged for 5 min at 16,000 × *g.* The supernatant fraction was collected as the cytoplasmic extract. The insoluble pellet fraction was resuspended in 100 μl of nuclear extraction reagent and incubated for 40 min on ice. The suspension was then centrifuged for 10 min at 16,000 × *g*, and the supernatant was collected as the nuclear extract. The amounts of NP, GAPDH, and LaminB1 in the nuclear or cytoplasmic fraction were determined by Western blotting with a rabbit anti-NP pAb, a rabbit anti-GAPDH pAb, and a rabbit anti-LaminB1 pAb, respectively.

### Confocal microscopy.

A549 cells treated with *FFAR2* siRNA (30 nM), 4-CMTB (100 μM), Cmp58 (10 μM), or DMSO in glass-bottom dishes were infected with AH05 (H5N1) virus at an MOI of 5. At 2, 3, or 4 h p.i., cells were fixed with 4% PFA for 30 min and permeabilized with 0.5% Triton X-100 in PBS for 15 min. The permeabilized cells were blocked with 5% BSA in PBS for 1 h and then incubated with the rabbit anti-NP pAb for 1 h. The cells were washed three times with PBS and incubated with Alexa Fluor 633 goat anti-rabbit IgG(H+L) for 1 h. After three washes, the cells were incubated with DAPI (4′,6-diamidino-2-phenylindole; Thermo Fisher Scientific) for 15 min to stain the nuclei. Images were acquired by using the LSM 800 confocal microscope with Airyscan (Zeiss, Oberkochen, Germany).

### Dual-luciferase reporter assay.

A luciferase assay to determine viral RNP activity was performed as described previously (67, 68). Briefly, HEK293T cells were transfected with *FFAR2* siRNA or scrambled siRNA (30 nM) for 24 h, followed by cotransfection with four viral RNP complex expression plasmids from WSN virus (pCAGGS-PB2, pCAGGS-PB1, pCAGGS-PA, and pCAGGS-NP [0.5 μg of each]), the construct pHH21-SC09NS F-Luc (0.2 μg), and an internal control, pRL-TK (0.01 μg). At 36 h posttransfection, HEK293T cells were split by using the dual-luciferase reporter assay system (Promega), and the luciferase activities were measured on a GloMax 96 microplate luminometer (Promega).

HEK293T cells were treated with *FFAR2* siRNA or scrambled siRNA (30 nM) for 24 h and then transfected with the ISRE-Luc reporter plasmid (0.2 μg) and the pRL-TK control plasmid (0.01 μg) for 24 h. The luciferase activity of the transfected cells was determined by performing a dual-luciferase reporter assay.

### Mx1 expression stimulated by IFN-α or IFN-β.

A549 cells grown in 12-well plates were transfected with *FFAR2* siRNA or scrambled siRNA (30 nM) for 48 h and then left untreated or treated with 100 U/ml of IFN-α (Sigma-Aldrich) or 25 pg/ml of IFN-β (R&D Systems, Minneapolis, MN) for 24 h. Cell lysates were subjected to Western blotting with a rabbit anti-Mx1 pAb to determine the level of Mx1 protein expression.

### Analysis of sialic acid receptor.

A549 cells were transfected with *FFAR2* siRNA or scrambled siRNA (30 nM). After 48 h, the cells were trypsinized, fixed with 4% PFA for 20 min, and then stained with an Alexa Fluor 488 conjugate of wheat germ agglutinin (WGA) (Invitrogen), a commonly used agent for the detection of sialic acids (SAs) on the cell surface. After two washes with PBS, the cell suspensions were subjected to flow cytometry on a FACSAria flow cytometer (BD Biosciences, Franklin Lakes, NJ). In a separate experiment, A549 cells treated with siRNA and fixed with PFA were stained with biotinylated Maackia amurensis lectin (MAL) (α-2,3-SA) or Sambucus nigra lectin (SNA) (α-2,6-SA) (Vector Laboratories, Burlingame, CA). The bound biotinylated lectins were captured by streptavidin conjugated with Cy-5 (Life Technologies). After three washes, the cell suspensions were subjected to flow cytometry on a FACSAria flow cytometer (BD Biosciences). A549 cells pretreated with neuraminidase (Sigma-Aldrich) at 37°C for 2 h were stained with WGA, MAL, or SNA and used as negative controls. The data were analyzed by using FlowJo software (FlowJo, Ashland, OR).

### Virus attachment assay.

A549 cells in 6-cm dishes were treated with siRNA targeting *FFAR2* or with scrambled siRNA (30 nM) for 48 h and then infected with AH05 (H5N1) virus (MOI = 5) on ice at 4°C for 1 h. The cells were trypsinized, fixed with 4% PFA for 30 min, and then stained with the corresponding primary (mouse anti-NP mAb, permeabilized with 0.5% Triton X-100, and mouse anti-HA mAb, without permeabilization) and secondary [Alexa Fluor 488 goat anti-mouse IgG(H+L)] antibodies. After three washes with PBS, the cell suspensions were subjected to flow cytometry on a FACSAria flow cytometer (BD Biosciences). The data were analyzed by using FlowJo software.

### IAV internalization assay.

A549 cells were grown in 6-well plates to 90% confluence and then infected with AH05 (H5N1) virus (MOI = 5) for 1 h on ice at 4°C. The infected cells were washed five times with ice-cold PBS (pH 7.2) or PBS-HCl (pH 1.3). Afterwards, the cells were lysed with SDS-PAGE loading buffer (Solarbio, Beijing, China) and then subjected to Western blotting with a rabbit anti-NP pAb.

A549 cells in 6-well plates were treated with siRNA targeting *FFAR2* or *AP2B1* or with scrambled siRNA (30 nM) for 48 h and then infected with AH05 (H5N1) virus (MOI = 5) on ice at 4°C for 1 h, followed by a culture temperature shift to 37°C for 30 min to allow for internalization. The cells were then washed five times with ice-cold PBS-HCl (pH 1.3) to remove the attached but not-yet-internalized virions and then subjected to Western blotting with a rabbit anti-NP pAb.

A549 cells were grown in 6-well plates to 90% confluence and then treated with DMSO, 4-CMTB (100 μM), or Cmp58 (10 μM) for 3 h at 37°C prior to infection with AH05 (H5N1) virus (MOI = 5) at 37°C. The cells were washed five times with ice-cold PBS-HCl (pH 1.3) at the indicated time points p.i. to remove the attached but not-yet-internalized virions and then subjected to Western blotting with a rabbit anti-NP pAb.

### Bimolecular fluorescence complementation assay.

HeLa cells grown in 96-well plates were transfected with the indicated pair of VN- and VC-tagged constructs by using Lipofectamine LTX and Plus reagents. pCAGGS-VN/pCAGGS-VC was used as a negative control (69). At 36 h posttransfection, cells were fixed with 4% PFA for 30 min and then incubated with Hoechst 33342 to stain the nuclei. Images were captured by using the Operetta high-content imaging system.

### Co-IP experiment.

HEK293T cells grown in 6-well plates were transfected with the indicated plasmids by using the Lipofectamine LTX and Plus reagents. At 36 h posttransfection, cells were washed once with ice-cold PBS (pH 7.2) and lysed with IP lysis buffer (25 mM Tris-HCl [pH 7.4], 150 mM NaCl, 1% NP-40, 1 mM EDTA, 5% glycerol) (Pierce, Rockford, IL) containing a complete protease inhibitor cocktail (Roche Diagnostics GmbH, Mannheim, Germany) and PMSF for 30 min on ice. The supernatants were then immunoprecipitated with glutathione magnetic agarose beads (Pierce) or with a rabbit anti-GST pAb and protein A/G-agarose (Abmart, Berkeley Heights, NJ) and rocked at 4°C overnight. The beads were then washed twice with ice-cold PBS, and the bound proteins were separated by SDS-PAGE and detected by Western blotting.

### Western blotting.

Protein samples fractionated by SDS-PAGE were transferred onto nitrocellulose membranes (GE Healthcare). Membranes blocked with 5% skim milk in PBS were incubated overnight at 4°C with the appropriately diluted primary antibody in PBS containing 0.5% BSA. After incubation with DyLight 800 goat anti-rabbit IgG(H+L) and DyLight 800 goat anti-mouse IgG(H+L), blots were visualized by using an Odyssey CLX infrared imaging system (Li-Cor BioSciences, Lincoln, NE).

### Statistical analysis.

Statistical significance was determined by using Student’s two-tailed unpaired *t* test or analysis of variance (ANOVA) with GraphPad Prism software (GraphPad, San Diego, CA); *P* values of ≤0.05 were considered significant.
